# The Prospects of an Active Vaccine Against Asthma Targeting IL-5

**DOI:** 10.3389/fmicb.2018.02522

**Published:** 2018-10-24

**Authors:** Martin F. Bachmann, Aadil El-Turabi, Antonia Fettelschoss-Gabriel, Monique Vogel

**Affiliations:** ^1^Department of Immunology, RIA, University Hospital Bern, Bern, Switzerland; ^2^Department of BioMedical Research, University of Bern, Bern, Switzerland; ^3^The Jenner Institute, Nuffield Department of Medicine, The Henry Wellcome Building for Molecular Physiology, University of Oxford, Oxford, United Kingdom; ^4^Department of Dermatology, University Hospital Zurich, Zurich, Switzerland; ^5^Evax AG, Münchwilen, Switzerland

**Keywords:** virus-like particle, IL-5, vaccine, asthma, cytokine, vaccination

## Abstract

Allergen-specific T helper type 2 (Th2) responses followed by eosinophilic inflammation of the lung are important causes of allergic asthma. Interleukin-5 (IL-5) is a master regulator of eosinophil differentiation as well as activation. Blocking IL-5 using monoclonal antibodies (mAbs) against IL-5 is a powerful way to improve asthmatic symptoms in patients with an eosinophilic component of the disease. We have previously shown that vaccination against IL-5 can abrogate eosinophilic inflammation of the lung in allergic mice. More recently, we have demonstrated that eosinophil-mediated skin disease in horses with insect bite hypersensitivity can be strongly reduced by vaccination against IL-5. Here we would like to propose the development of a similar vaccine for the treatment of asthma in humans.

## Asthma – a Significant Chronic Respiratory Disease That Represents a Growing Challenge

Asthma is a globally significant chronic respiratory disease with upward of 300 million patients experiencing considerable morbidity ranging from occasional breathing difficulties (shortness of breath, coughing, wheezing, and chest tightening) to more severe exacerbations (asthma attack) requiring clinical interventions (Network, 2018). Inflammation in the lungs can be triggered by a variety of non-specific stimuli such as cold air and exercise, as well as by allergens or infectious microorganisms such as viruses. In turn, these events can activate a combination of innate and adaptive immune responses and cellular inflammation, resulting in symptoms like bronchial smooth muscle hyper-reactivity, excess mucus production from goblet cells within epithelia, and potential for bronchial remodeling and airway narrowing. Given the remarkable heterogeneity in both stimuli and responses, it is not surprising to see the involvement and overlap of several distinct soluble mediators and cell types. Key amongst these are cytokines involved in immunoglobulin isotype switching (IgE synthesis by IL-4), mast cell proliferation (IL-9), airway hyper-responsiveness and mucus production (IL-13), and eosinophil inflammation (IL-5). In addition to type 2 T helper cell (Th2) allergic responses, also Th1, Th17, and Th9 cellular subsets have been implicated (Holgate and Polosa, 2008; Lambrecht and Hammad, 2015).

In many patients, the disease can be controlled by a combination of anti-inflammatory immunosuppressants (such as inhaled corticosteroids) and by bronchodilators that relax constricted airway smooth muscle (e.g., β_2_-adrenergic agonists). However, between 5–10% of patients are refractory to corticosteroid treatments thus complicating management of their asthma and often leading to serious exacerbations requiring hospital intervention. This has encouraged the development of several biological drug alternatives based on monoclonal antibodies (mAbs) for those individuals where conventional small molecule drugs have failed.

## mAbs Have Changed the Way We Treat Chronic Diseases by Allowing the Targeting of Protein-Protein Interactions Outside the Cell

Generation of mAbs was described more than 40 years ago in mice (Kohler and Milstein, 1975), and over the past 20 years, the technology has started to change clinical practice (Cui et al., 2017). In contrast to small molecular drugs, which classically target intracellular proteins, enzymes, ion channels, and G-coupled proteins, mAbs have allowed the blocking of extracellular protein-protein interactions. They may inhibit receptor–ligand interactions or specifically target cell surface receptors. Blocking protein–protein interactions with high specificity has made it possible to inhibit cytokines, chemokines and growth factors implicated in otherwise intractable disease conditions while targeting cell surface receptors is often followed by antibody-dependent cellular cytotoxicity (ADCC) and is generally used for the elimination of cancer cells. Due to these unique properties, mAbs have become the most rapidly growing revenue source for the pharmaceutical industry. The top-in-class antibody Humira^®^ was the world’s best-selling drug 2015 with annual global expenditure reaching US$14 billion. Moreover, 6 of the 10 biggest blockbuster drugs are mAbs^1^.

The flip-side of the argument is the high costs that are associated with the generation of these biomolecules, cutting a large fraction of the world’s population off from the benefits brought by this new treatment modality. In addition, despite mAbs high efficiency, treatment involves injections of very substantial amounts of protein that may cause local side-effects such as pain and irritation or even severe anaphylactic reactions. An additional potential issue associated with the use of mAbs is the induction of anti-drug antibodies that may neutralize or eliminate the injected mAbs and may accelerate local or systemic adverse events during injection (Steenholdt et al., 2011, 2012).

Many of these problems could be tackled by moving on from the current passive vaccination strategies with mAbs to active vaccination approaches that instruct the body to generate its own antibody responses. Potential issues with this new approach are that it may be difficult to (i) reach sufficient levels of antibodies and (ii) the response may be less controllable; in particular, the potential irreversibility of the induced antibody responses is an obvious issue. However, recent developments in vaccine design, genetics, and clinical research are converging to pave the way for the clinical development of such auto-vaccines. It was recently shown that anti-cytokine vaccination could reach critical levels in humans (Lauwerys et al., 2014; Cavelti-Weder et al., 2016; Ducreux et al., 2016) and other target species (Bachmann et al., 2018; Fettelschoss-Gabriel et al., 2018b), and that the induced antibody responses are indeed reversible. The present review will examine recent findings and outline how these findings may be applied to the development of a vaccine against asthma targeting IL-5.

## Harnessing the Immune System: How to Induce Self-Specific Antibody Responses Directed Against Cytokines

**FIGURE 1:**
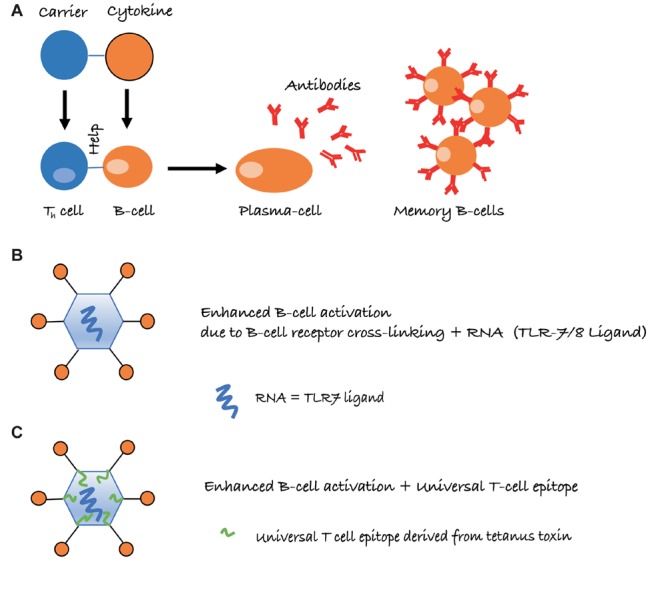
Schematic representation of intermolecular help for the induction of cytokine-specific antibody responses. **(A)** Cytokines are conjugated to a carrier protein, resulting in cytokine-specific B cells receiving, however, carrier specific T-cell help. **(B)** Conjugation to virus-like particles (VLPs) further increases antibody responses, due to repetitive display of the antigen and packaged non-coding RNA, which is a ligand for toll-like receptor 7/8 (TLR7/8) and present in many RNA virus-derived VLPs. **(C)** Presence of a universal T cell epitope may further improve Th cell dependent IgG responses, in particular if the epitope is derived from tetanus toxin (TT), as essentially everybody harbors memory Th cells against TT.

Long-lived IgG responses require activation of specific B as well as (follicular) Th cells. However, the immune system is usually tolerant to self-proteins, and induction of cytokine-specific antibody responses, therefore, requires overcoming or bypassing B cell as well as Th cell tolerance. In general, soluble proteins do not cause B cell tolerance and antibody responses against soluble self-proteins are usually controlled by Th cell tolerance only (Adelstein et al., 1991). As a case in point, we have recently shown that introduction of a single virus-derived Th cell epitope into IL-1β was able to overcome B cell unresponsiveness and immunization with this modified cytokine formulated in adjuvants readily resulted in strong and specific IgG responses (Spohn et al., 2014). Hence, linking cytokines to a source of Th cell epitopes is able to drive specific B cell responses. This is similar to classical carbohydrate conjugate vaccines where B cells recognize the carbohydrate and Th cells recognize the carrier protein to which the carbohydrates are linked (Mond et al., 1995) (Figure 1A).

Conventional protein-cytokine conjugate vaccines have struggled to induce sufficient levels of antibodies to reach clinical efficacy (Ratsimandresy et al., 2009). Indeed, while the use of strong adjuvants in preclinical experiments may help to increase the efficiency in murine model diseases, translation to the clinic is difficult, as such adjuvants cannot be used in humans. A case in point is a vaccine against TNF (Dalum et al., 1999), which successfully treated rheumatoid arthritis in mice but failed to reach efficacy in humans. One reason for this failed translation was the use of complete Freund’s adjuvants (CFA) in the murine experiments, an adjuvant that under no circumstances could be used in humans, due to the induction of local granuloma or systemic reactogenicity, hypersensitization to mycobacteria (tuberculin) and heightened risk of autoimmune disease.

Virus-like particles (VLPs) may offer an elegant solution to this problem. By displaying cytokines on the surface of VLPs, it is possible to induce high levels of neutralizing antibodies without the use of adjuvants. Several factors account for the high immunogenicity of antigens displayed on VLPs. One important parameter is the repetitive display of the antigen, causing efficient cross-linking of B cell receptors (BCRs) on B cells, constituting a strong activation signal (Figure 1B). Also, repetitive epitopes efficiently recruit components of the innate humoral immune system in particular complement and natural IgM (Bachmann and Jennings, 2010). This enhances B cell activation via CD21 and facilitates B cell-mediated antigen deposition on follicular dendritic cells (FDCs), which present native antigen to B cells (Link et al., 2012). Antigen on FDCs is essential for the formation of germinal centers, which in turn are essential for the generation of memory B and long-lived plasma cells (Liu et al., 1992). Hence, the ability to recruit natural IgM and complement greatly increases immunogenicity. An additional reason for the strong immunogenicity of antigens displayed on VLPs is the bacterial RNA packaged by many VLPs produced in bacteria. RNA is a ligand for TLR7/8 and serves as “packaged adjuvant” enhancing IgG as well as IgA responses (Jegerlehner et al., 2007; Bessa et al., 2009; Hou et al., 2011) (Figure 1B). For this reason, cytokines on the surface of VLPs can induce strong and neutralizing antibody responses that show preclinical and clinical efficacy. As an example, formulation of an IL-1β-VLP conjugate vaccine in Alum was sufficient to induce neutralizing anti-IL-1 antibodies in at least a fraction of human type II diabetes patients (Cavelti-Weder et al., 2016). We have recently optimized the immunogenicity of a VLP derived from the plant-specific cucumber mosaic virus. By introducing a universal T cell epitope derived from tetanus toxin, we generated a VLP optimized for the elderly (CuMV_TT_) (Zeltins et al., 2017) since essentially all individuals have been immunized multiple times against tetanus and harbor specific memory Th cells (Figure 1C). Using this CuMV_TT_, we have been able to treat atopic dermatitis in dogs and horses by immunizing against IL-31 and IL-5, respectively (Bachmann et al., 2018; Fettelschoss-Gabriel et al., 2018b). Hence, cytokines displayed on VLPs hold promise to induce clinically relevant antibody levels for the treatment of chronic diseases.

It is important to note that maintenance of T cell tolerance for self-antigens is key, so vaccines targeting self-molecules should avoid breaking tolerance completely. Otherwise, there may be a risk of undesired auto-immune complications. An important safety aspect of VLP-based vaccines is that they confer self-antigens with repetitive presentation on their surface [an immunogenic feature of many pathogens, also called pathogen-associated structural pattern (PASP) (Bachmann and Jennings, 2010)]. Hence, VLPs place the self-antigen in an immunologically active context and link it to T-cell help directed toward the VLP carrier, features that are absent from endogenous self-antigens (see Figure 1). Therefore, the B cells receive bystander T cell help for the self-antigen only when coupled to the VLP, whereas uncoupled self-antigens will not receive bystander T cell help. Reassuringly in preclinical studies for psoriasis (a chronic inflammatory skin disorder), a VLP-vaccine targeting the pro-inflammatory cytokine IL-17, we demonstrated that induced anti-IL-17 antibody levels reversed after cessation of immunization and stimulating an endogenous flare of the cytokine using imiquimod (a TLR7/8 ligand and potent IL-17 activator) was unable to influence antibody responses, as T cell help from the VLP was missing (Rohn et al., 2006; Zeltins et al., 2017). This is fundamentally different from classical prophylactic vaccines that are designed to trigger the immune system to respond upon re-encountering the target pathogen.

## Asthma and the Role of Il-5

Allergic asthma is characterized by chronic inflammation of the airways and manifests as episodes of airway obstruction and wheezing. Roughly half of severe asthma clinical cases appear to present with a non-allergic etiology. However, a key feature for a subset of asthma patients is increased levels of granulocytes called eosinophils, which are very toxic cells in the body (Yancey et al., 2017). Chronic eosinophilic airway inflammation is a particularly severe form of asthma, which eventually causes structural changes of the airways. This disease progression is summarized as airway remodeling that may result in bronchial hyperresponsiveness to non-specific stimuli, a characteristic of intrinsic (non-allergic) asthma (Cohn et al., 2004).

Standard therapies for asthma are non-specific and involve corticosteroids and long- or short-acting β-adrenoceptor agonists. These medications, however, only suppress symptoms but fail to treat the underlying cause of the disease. Furthermore, compliance is generally low, in particular for twice-daily inhalation regimens (Bender et al., 1997; Onyirimba et al., 2003). Non-specific immunotherapy using VLPs loaded with a ligand for TLR9 is a promising option for the treatment of asthma but requires further investigation (Beeh et al., 2013; Rank et al., 2013). In contrast, specific immunotherapy (SIT) represents a potential disease-modifying therapy that is, as the name implies, more specific than the classical drugs (Nagata and Nakagome, 2010). SIT, however, is only possible for a limited number of allergens, time-consuming and cannot be performed with patients suffering from severe asthma (Creticos, 1992; Walker et al., 1995). Drugs specifically interfering with the allergic immune response are promising new alternatives. A key cell type in asthma is the allergen-specific Th2 cells, which produce IL-4, causing generation of IgE followed by activation of mast cells. In addition, Th2 cells release IL-13, increasing obstructing mucus production and IL-5, as well as eotaxin, recruiting eosinophils. An alternative to the non-specific standard therapy with corticosteroids or SIT, is therefore to specifically target the cells and factors involved in asthma. Four such FDA approved drugs are mAbs against IgE (Omalizumab), IL-5 (Mepolizumab, Reslizumab) (Quirce et al., 2017), and recently IL-5 receptor (Benralizumab) (Kupczyk and Kuna, 2018). Omalizumab neutralizes IgE and may even remove it from mast cells. Hence, blocking IgE mostly results in reduced mast cell activity, most important during the early asthma response. In contrast, therapies blocking IL-5 aim at reducing blood and tissue eosinophils thereby being more potent at halting later phases of the response, including airway remodeling. Mepolizumab, Reslizumab, and Benralizumab are therefore mostly used for the treatment of severe eosinophilic asthma, a chronic condition requiring life-long treatment. GlaxoSmithKline (GSK) is currently negotiating with the authorities for approval of Mepolizumab in chronic obstructive pulmonary disease (COPD) subsequent to a successful phase III study in the indication (Pavord et al., 2017). Nonetheless, adverse reactions such as urticaria, anaphylaxis or serum sickness have been reported with passive administration of antibodies and repeated injections are required to maintain effectiveness. With an excellent safety profile (Leung et al., 2017) and the requirement for long-term if not life-long treatment, IL-5 might be an ideal target for the development of an active vaccine against asthma and potentially COPD.

## Preclinical and Clinical Data Supporting the Development of a Vaccine Against Il-5

IL-5 is a soluble low abundant protein and tolerance at the B cell level may therefore not be expected (Bachmann and Dyer, 2004). Indeed, the coupling of IL-5 to VLPs derived from the bacteriophage Qβ readily induced strong and neutralizing antibody responses in mice in the absence of an adjuvant (Zou et al., 2010). Neutralizing antibodies were able to reduce numbers of eosinophils in peripheral blood and reduced eosinophil infiltration in the lung of asthmatic mice by more than 95%. Hence, vaccination against IL-5 resulted in protection against eosinophilic infiltration, the same principal biological effect observed for commercial anti IL-5 antibody therapies. Similar observations have been made with a DNA based vaccine (Hertz et al., 2001). DNA vaccines, however, are usually not immunogenic in humans and therefore difficult to translate into clinical application. More recently, we have demonstrated that VLP-based vaccines are efficacious in a more real-life setting, namely insect bite hypersensitivity (IBH) in horses. IBH is an allergic response to bites of midges (*Culicoides*) and results in strong inflammation of the skin by eosinophils. Similarly as seen in allergic asthma in humans (Price et al., 2016), blood eosinophil counts correlated with disease severity (Fettelschoss-Gabriel et al., 2018b). In a double-blind, placebo-controlled study, IBH horses were immunized against IL-5 using a conjugate vaccine consisting of IL-5 conjugated to the immune optimized CuMV_TT_-VLP (Zeltins et al., 2017). IBH is caused by insect-bites and therefore seasonal with no symptoms during the winter. Horses were vaccinated with IL-5-CuMV_TT_ in the absence of adjuvants before and during the season. The vaccine was well tolerated with no signs of adverse events after repeated doses, and no difference in helminth burden could be detected. Strikingly, symptoms of IBH in immunized horses were strongly reduced compared to the previous untreated season of the same horses as well as to the placebo horses (Fettelschoss-Gabriel et al., 2018b). Anti-IL-5 responses declined in all horses with defined half-lives of a few months and thus were reversible. More recently, we have shown that horses can be immunized over multiple seasons and that protection against IBH remains stable (Fettelschoss-Gabriel et al., 2018a). Hence, vaccination of horses against IL-5 results in strong protection against IBH, an eosinophil-mediated inflammatory disease. By analogy, we are confident that a human version of the vaccine will protect against severe asthma, with an eosinophil-mediated pathology for the disease of the lung.

## Conclusion

IL-5 is an excellent target for the treatment of allergic and non-allergic asthma with an eosinophilic component. We propose to develop a vaccine against IL-5 for the treatment of asthma in humans to broaden patient access to the highly effective therapy and to facilitate long-term treatment.

## Author Contributions

Each author contributed to the writing of the manuscript.

## Conflict of Interest Statement

MB and AF-G are involved companies developing vaccines based on virus-like particles. The remaining authors declare that the research was conducted in the absence of any commercial or financial relationships that could be construed as a potential conflict of interest.

## Footnotes

1 https://labiotech.eu/top-10-best-selling-biologicals-blockbusters-2015/
